# Electric Cell-Substrate Impedance Sensing To Monitor Viral Growth and Study Cellular Responses to Infection with Alphaherpesviruses in Real Time

**DOI:** 10.1128/mSphere.00039-17

**Published:** 2017-04-05

**Authors:** Matthew R. Pennington, Gerlinde R. Van de Walle

**Affiliations:** Baker Institute for Animal Health, College of Veterinary Medicine, Cornell University, Ithaca, New York, USA; Northwestern University Feinberg School of Medicine

**Keywords:** ECIS, alphaherpesvirus, antiviral agents, genome editing, growth kinetics

## Abstract

Electric cell-substrate impedance sensing (ECIS) measures changes in an electrical circuit formed in a culture dish. As cells grow over a gold electrode, they block the flow of electricity and this is read as an increase in electrical impedance in the circuit. ECIS has previously been used in a variety of applications to study cell growth, migration, and behavior in response to stimuli in real time and without the need for cellular labels. Here, we demonstrate that ECIS is also a valuable tool with which to study infection by alphaherpesviruses. To this end, we used ECIS to study the kinetics of cells infected with felid herpesvirus type 1 (FHV-1), a close relative of the human alphaherpesviruses herpes simplex virus 1 (HSV-1) and HSV-2, and compared the results to those obtained with conventional infectivity assays. First, we demonstrated that ECIS can easily distinguish between wells of cells infected with different amounts of FHV-1 and provides information about the cellular response to infection. Second, we found ECIS useful in identifying differences between the replication kinetics of recombinant DsRed Express2-labeled FHV-1, created via CRISPR/Cas9 genome engineering, and wild-type FHV-1. Finally, we demonstrated that ECIS can accurately determine the half-maximal effective concentration of antivirals. Collectively, our data show that ECIS, in conjunction with current methodologies, is a powerful tool that can be used to monitor viral growth and study the cellular response to alphaherpesvirus infection.

**IMPORTANCE** Alphaherpesviruses, including those that commonly infect humans, such as HSV-1 and HSV-2, typically infect and cause cellular damage to epithelial cells at mucosal surfaces, leading to disease. The development of novel technologies to study the cellular responses to infection may allow a more complete understanding of virus replication and the creation of novel antiviral therapies. This study demonstrates the use of ECIS to study various aspects of herpesvirus biology, with a specific focus on changes in cellular morphology as a result of infection. We conclude that ECIS represents a valuable new tool with which to study alphaherpesvirus infections in real time and in an objective and reproducible manner.

## Abstract (precis)

Alphaherpesviruses, including those that commonly infect humans, such as HSV-1 and HSV-2, typically infect and cause cellular damage to epithelial cells at mucosal surfaces, leading to disease. The development of novel technologies to study the cellular responses to infection may allow a more complete understanding of virus replication and the creation of novel antiviral therapies. This study demonstrates the use of ECIS to study various aspects of herpesvirus biology, with a specific focus on changes in cellular morphology as a result of infection. We conclude that ECIS represents a valuable new tool with which to study alphaherpesvirus infections in real time and in an objective and reproducible manner.

## INTRODUCTION

Electric cell-substrate impedance sensing (ECIS) is a label-free, impedance-based method used to study cellular kinetics in real time and relies on measurement of the changes in the electrical impedance of a circuit formed in a tissue culture dish plated with cells. This methodology was first described by Giaever and Keese (1) and is used to quantify morphological changes in a nanoscale range, well beyond the limits of light microscopy. Cells are cultured on small thin-film gold electrodes that are available for use in a variety of patterns and sizes, depending on the specific ECIS application. A noninvasive alternating current is then applied through a resistor over the kilohertz frequency range, and the change in electrical impedance is calculated at predetermined intervals (2). As cells attach to and spread out over the electrodes, the impedance increases because of the insulation of the electrodes by the cell membranes and the formation of tight junctions blocking the electrical current’s flow. Conversely, when cells are stressed and/or dying, the disruption of cell-to-cell junctions due to rounding of membranes and detachment of cells from the well plates allows greater electrical current passage, which is read as a decrease in impedance over time (3).

ECIS is well established in many fields, such as cancer metastasis, toxicology, wound healing, and other cell biology-related fields (4–6), and recently is also becoming increasingly recognized as a useful tool for virologic studies. Thus far, ECIS has been used primarily to study viral growth and cellular responses to infection with RNA viruses, such as respiratory syncytial virus, influenza A virus (IAV), and Sin Nombre virus (7–9). Additionally, one study described the use of ECIS to study the effects of transient overexpression of Kaposi’s sarcoma-associated herpesvirus proteins MIR-1 and MIR-2 on the attachment, spreading, and junction formation of immortalized dermal microvascular endothelial cells (10). However, ECIS has not yet been used to study the cellular responses to infection with herpesviruses in the context of a lytic infection.

Felid herpesvirus type 1 (FHV-1), a member of the *Alphaherpesvirinae* subfamily and a close relative of herpes simplex virus 1 (HSV-1) and HSV-2, the causative agents of cold sores and genital herpes, respectively, is an important pathogen of cats worldwide. FHV-1 infection is not only the single most important pathogen contributing to feline upper respiratory infection, but it is also the most common viral pathogen to cause ocular disease in cats (11, 12). FHV-1 replication in the cornea of the eye generally results in conjunctivitis, but other clinical symptoms, including corneal ulceration and the development of chronic stromal keratitis, are also very common (11–13). Likewise, HSV-1 can cause serious ocular disease in humans, and since FHV-1 ocular infection closely mimics all aspects of the clinical presentation of the disease and the associated immune responses seen in humans, cats are accepted as an excellent comparative model species for ocular HSV-1 infection (14).

In the present study, we used FHV-1 as a proof of concept to evaluate the potential of ECIS to study viral growth in and cellular responses to infection with alphaherpesviruses as a complement to the conventionally used infectivity assays. We found that ECIS could detect dose-dependent changes in impedance due to virus-induced cell death at various multiplicities of infection (MOIs) with FHV-1. Moreover, we showed that ECIS can be used to characterize the growth of recombinant herpesviruses and is a useful tool with which to accurately calculate the half-maximal effective concentration (EC_50_) of antivirals.

## RESULTS

### ECIS can be used to model kinetic growth curves and to study morphological changes in response to herpesvirus infection.

An essential preliminary step when using ECIS to ensure proper analysis and interpretation of the data is to determine the optimal frequencies at which to monitor changes in the impedance (Z), resistance (R), and capacitance (C) of cells, as optimal frequencies can vary, depending on the cell type. The optimal frequencies are those at which the largest difference between wells that contain cells and wells that do not contain cells (cell free) is measured. We used the Crandell-Rees feline kidney (CRFK) cell line in our studies and determined, by using the ECIS software, the optimal frequency for impedance to be 16,000 Hz, that for resistance to be 4,000 Hz, and that for capacitance to be 64,000 Hz (see Fig. S1A in the supplemental material). These values were in agreement with the suggested defaults, as determined by Applied BioPhysics (Troy, NY), and were used for all subsequent data acquisitions.

10.1128/mSphere.00039-17.1 FIG S1  Additional data for validation of ECIS as a tool with which to monitor alphaherpesvirus infections. (A) Graphs of the ratios of impedance (Z), resistance (R), and capacitance (C) of CRFK-containing wells to those of cell-free wells at 24 hpp, calculated and plotted as a function of frequency. The vertical broken blue line in each graph represents the frequency with the maximal difference between cell-containing and cell-free wells. (B) Light microscopy pictures of ECIS wells infected with FHV-1 at the MOIs indicated at different time points (0 to 48 hpi). Pictures in colored boxes represent the time points at which a substantial CPE was observed at the different MOIs. The black bars in the images are ECIS electrodes. Scale bar, 25 µm. Download FIG S1, PDF file, 0.4 MB. Copyright © 2017 Pennington and Van de Walle. 2017 Pennington and Van de Walle This content is distributed under the terms of the Creative Commons Attribution 4.0 International license.

**FIG 1:**
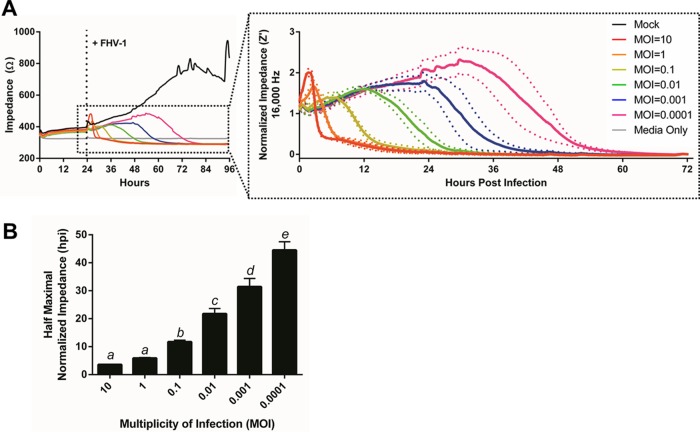
ECIS easily distinguishes between wells of cells infected with different amounts of FHV-1. (A) CRFK cells were plated in ECIS wells, and impedance was monitored for 24 h at 16,000 Hz. Wells were then infected with FHV-1 at the MOIs indicated. The inset shows normalized impedance (Z′) values following FHV-1 infection. (B) Half-maximal Z′ (Z′_50_) values for impedance curves based on data from panel A. Different letters indicate significantly different Z′_50_ values, as determined by one-way ANOVA.

Initially, we evaluated whether ECIS could discriminate between wells of cells infected with FHV-1 at various MOIs. To this end, CRFK cells were plated onto an ECIS polyethylene terephthalate 96-well plate with 10 interdigitated electrode fingers (96W10idfPET) and impedance was monitored at 16,000 Hz for an initial 24 h to establish baseline impedance levels (Fig. 1A). At 24 h postplating (hpp), cells were infected with 10-fold serial dilutions of FHV-1 and impedance was measured until the impedance of all infected wells reached that of the cell-free control wells, which corresponded to 72 or 96 h postinfection (hpi). All of the wells initially showed a peak in impedance immediately following virus or control medium addition, most likely as a result of the physical manipulation of the cells, although the height of this peak varied with the MOI. Such a peak is commonly observed in ECIS experiments and has been reported previously in the context of IAV infection (8), despite morphological changes not being readily observable by light microscopy at this time (data not shown). All of the wells with FHV-1-infected cells then showed a subsequent decrease in impedance, which occurred at different points postinfection, with wells infected with higher MOIs having an earlier decrease than wells infected with low MOIs (Fig. 1A). These changes in impedance corresponded to the formation of a cytopathic effect (CPE), characterized by rounding of cell membranes and detachment of the cells from the electrodes, as observed by light microscopy performed at specific time points (see Fig. S1B). In contrast, the impedance of wells with mock-infected cells continued to rise steadily over time, corresponding to the formation of a confluent monolayer. At approximately 72 hpp, the impedance in the mock-infected wells began to plateau and oscillate because of micromotion of the wells on the electrode, as has been reported previously in ECIS experiments (Fig. 1A) (15). As the impedance at the time of infection varied slightly between replicates because of minor differences in cell numbers, we chose to normalize the Z′ impedance data for further analysis (Fig. 1A, inset). These normalized data were then used to calculate the time point postinfection at which each MOI induced a half-maximal drop in the normalized impedance, and this was termed the Z′_50_ time point. We found no statistically significant difference between the Z′_50_ values, expressed in hours postinfection, at MOIs of 10 and 1, but all subsequent 10-fold serial dilutions of FHV-1 did show statistically significant differences in Z_50_ values (Fig. 1B).

**FIG 2:**
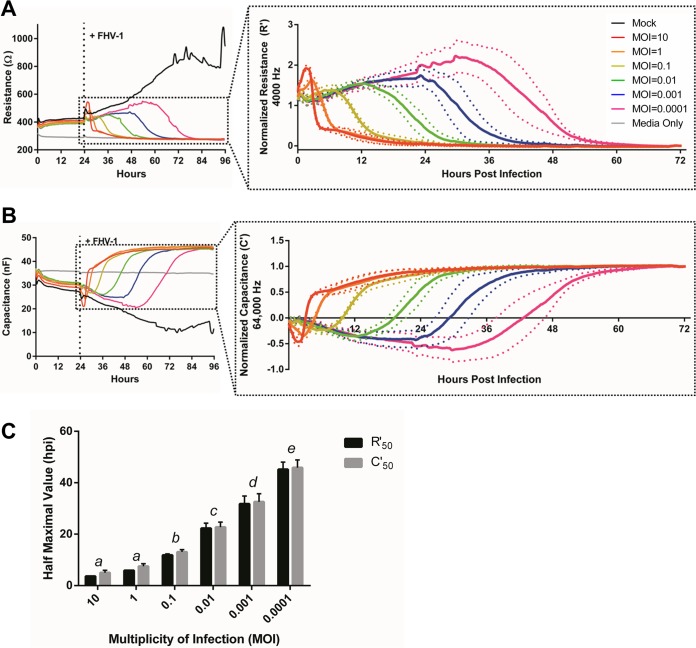
FHV-1 infection induces dose-dependent changes in resistance and capacitance (A, B). Resistance (R) and capacitance (C) measurements at 4,000 and 64,000 Hz, respectively, following infection of CRFK cells with FHV-1 at the MOIs indicated at 24 hpp. The insets show normalized resistance (R′) and capacitance (C′) values following FHV-1 infection. (C) Comparison of half-maximal R′ (R′_50_) and C′ (C′_50_) values based on data from panels A and B. Different letters indicate MOI R′_50_ or C′_50_ values that are significantly different, as determined by one-way ANOVA. No significant differences between R′_50_ and C′_50_ values were observed at any MOI, as determined by Student *t* test.

The ECIS ZƟ instrument we used in this study calculates complex impedance by using the measured resistance and capacitance parameters. Therefore, these values can also be analyzed individually to gain more biological information on how exactly cells respond to manipulation, such as infection with an alphaherpesvirus. Resistance is applied at low frequency, in our study, optimally at 4,000 Hz, to induce the electrical current to flow underneath and in the paracellular space between cells and thus provides information on the nature of tight junctions and other cell-cell interactions (16). Similar to impedance, we observed an MOI-dependent decrease in resistance in response to infection with FHV-1 (Fig. 2A), indicating that herpesvirus infections disrupt cell-cell interactions. This is in agreement with previous work showing that HSV-2 causes downregulation of gap junctions between infected cells (17). Capacitance, in contrast, is applied at high voltage, in our study, optimally at 64,000 Hz, and refers to the ability of the electrode in the well to store an electrical charge. The plasma membrane of the cell can act as a small capacitor, and as cells attach to the electrode, they restrict the ability of the electrode to store a charge. The capacitance of the circuit therefore decreases, as opposed to the observed increases in resistance and impedance, and represents cell attachment to and spreading over a substrate (16). The observed dose-dependent increase in capacitance upon infection with decreasing MOIs of FHV-1 (Fig. 2B) indicates that cell detachment from the electrodes takes longer when cells are infected with fewer viruses, as expected. When calculating both the half-maximal normalized resistance (R′_50_) and the half-maximal normalized capacitance (C′_50_) for each infectious dose (Fig. 2C), we observed the same statistically significant difference in dose responses to infection as seen in the Z′_50_ value (Fig. 1B, one-way analysis of variance [ANOVA], *P* ≤ 0.05). There was no statistically significant difference between the R′_50_ and C′_50_ values at any MOI (Student *t* test, *P* > 0.05) (Fig. 2C), indicating that disruption of cell-cell junctions and detachment of the cells from the electrode in response to FHV-1 infection occur at approximately the same time rather than sequentially.

Taken together, these results indicate that ECIS can distinguish between wells of cells infected with different amounts of FHV-1 and provide quantitative information about the morphological changes in infected cells in real time, without the need for static sample collection.

### ECIS can be used to characterize the growth kinetics of recombinant herpesviruses.

We recently decided to create fluorescently labeled recombinant FHV-1 for easy identification of infected cells by immunofluorescence and flow cytometry. To this end, we used CRISPR/Cas9 genome engineering, based on recently described protocols for editing HSV-1 (18–20), and fused the DsRed Express2 protein to the C terminus of glycoprotein (gD) on the basis of a previous study where introduction of monomeric red fluorescent protein at this location did not impact herpesvirus growth (21) (see Fig. S2A and B). We initially confirmed the location of the DsRed insertion by PCR analysis (see Fig. S2C) and Sanger sequencing. We subsequently characterized the growth of this FHV-1-gD-DsRed recombinant by using the traditional characterization assays such as viral plaque assays and single- and multistep growth kinetics compared to wild-type (WT) FHV-1.

10.1128/mSphere.00039-17.2 FIG S2  Creation, by CRISPR/Cas9 genome engineering, and characterization of FHV-1-gD-DsRed. (A) Schematic representation of the site of introduction of DsRed Express2 at the C-terminal end of *US6*, with the target site and sequence of the sgRNA indicated. (B) Schematic map of the pJET1.2-FHV-1-DsRed donor vector showing the FHV-1 homology regions to drive the insertion of DsRed via homology-directed repair. (C) Confirmation of DsRed Express2 insertion at the targeted location. Four primer sets were used to amplify different regions around the insertion site from WT and edited viruses. (D) CRFK cells were infected at 10 PFU/coverslip with FHV-1-gD-DsRed (red) or WT FHV-1 and stained with an anti-FHV-1-antibody (green), and nuclei were counterstained with DAPI (blue). (E) Quantification of the area of viral plaques showing the median value and quartiles. ****, *P* < 0.0001. Download FIG S2, PDF file, 0.3 MB. Copyright © 2017 Pennington and Van de Walle. 2017 Pennington and Van de Walle This content is distributed under the terms of the Creative Commons Attribution 4.0 International license.

**FIG 3:**
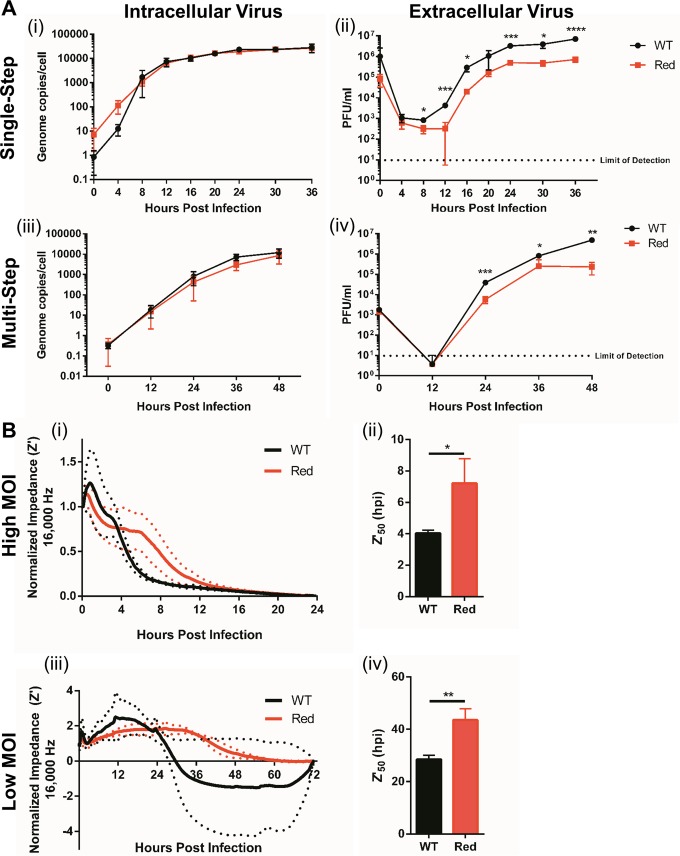
ECIS identifies the growth impairments of recombinant FHV-1. (A) CRFK cells were infected with FHV-1-gD-DsRed or WT FHV-1 at an MOI of 3 or 0.01 to measure single-step (graphs i and ii) or multistep (graphs iii and iv) growth curves, respectively, with conventional viral infectivity assays. qPCR was used to quantify intracellular genomic viral DNA copy numbers, and standard plaque assays were used to quantify extracellular virus titers. (B) Normalized impedance (Z′) values of CRFK cells infected with FHV-1-gD-DsRed or WT FHV-1 at a high MOI of 3 (i) or a low MOI of 0.01 (iii). Half-maximal Z′ (Z′_50_) values for impedance curves based on data from graphs i and iii were determined (graphs ii and iv, respectively). *, *P* < 0.05; **, *P* ≤ 0.01; ***, *P* ≤ 0.001; ****, *P* ≤ 0.0001.

When comparing the growth of FHV-1-gD-DsRed to the growth of WT FHV-1, we found no significant differences in intracellular viral genome copy numbers in the single-step growth experiments at any of the time points tested (Fig. 3A, graph i). However, we did observe an approximately 1-log reduction in extracellular infectious virus progeny in FHV-1-gD-DsRed-infected cells compared to WT-infected cells, which started at 8 hpi and remained for the duration of the experiment (Fig. 3A, graph ii). Similar patterns were also observed in the multistep growth experiments (Fig. 3A, graphs iii and iv). With growth-defective FHV-1-gD-DsRed in hand, we decided to evaluate whether ECIS is capable of detecting similar differences between FHV-1-gD-DsRed and WT FHV-1. To this end, wells with confluent monolayers of CRFK cells were infected with FHV-1 at low (matching the multistep kinetics) and high (matching the single-step kinetics) MOIs and impedance changes were monitored over time. At both low and high MOIs, a decrease in normalized impedance was observed at earlier time points in WT-infected wells compared to FHV-1-gD-DsRed-infected wells (Fig. 3B, graphs i and iii). Z′_50_ values were then calculated to statistically compare the ECIS results obtained with these two viruses, and we found significant differences in both high-MOI (single-step) experiments, with a half-maximal impedance at 4.0 ± 0.2 hpi in WT-infected wells compared to 7.2 ± 1.6 hpi in FHV-1-gD-DsRed-infected wells (Student *t* test, *P* = 0.03) (Fig. 3B, graph ii), and low-MOI (multistep) experiments, with a half-maximal impedance at 28.5 ± 1.6 hpi compared to 43.6 ± 4.2 hpi (Student *t* test, *P* = 0.004) (Fig. 3B, graph iv).

However, our current ECIS experiment does not allow us to determine the nature of the growth defect associated with fusion of DsRed to gD. To this end, we conducted conventional plaque size assays. We found that FHV-1-gD-DsRed produced significantly smaller plaques than WT FHV-1, suggesting that the recombinant virus is impaired in the ability to move from cell to cell (see Fig. S2D and E). DsRed has been reported to obligatorily tetramerize *in vivo* to form a rather rigid structure (22, 23), likely accounting for the defect in this viral protein.

Taken together, the results of these experiments indicate that ECIS is a useful tool with which to initially screen and identify differences between the replication kinetics of recombinant and WT alphaherpesviruses to allow a more targeted characterization of selected viruses by using conventional viral growth assays.

### ECIS can be used to calculate the EC_50_s of antivirals.

**FIG 4:**
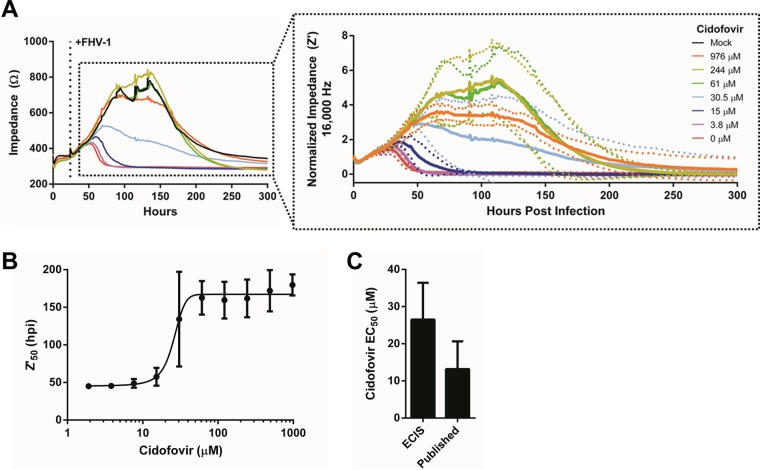
ECIS accurately determines the EC_50_ of the antiviral cidofovir. (A) CRFK cells were infected with MOI of 0.01 of FHV-1 and treated with 2-fold dilutions of cidofovir (ranging from 976 to 1.9 μM) at 24 hpp. Nontreated FHV1-infected and mock-infected CRFK cells were included as controls. The insert shows normalized impedance values (Z′) following FHV-1 infection and cidofovir treatment. For clarity, only Z′ values for a selected set of cidofovir concentrations are shown. (B) Dose-response curve of the Z′_50_ value of each cidofovir concentration used to determine the EC_50_. (C) Comparison of the EC_50_ of cidofovir as determined by ECIS in the present study to previously published EC_50_ values determined by standard plaque reduction assays (25–27). No significant difference between ECIS-based and previously published EC_50_s was observed.

Finally, we evaluated if ECIS could be used to calculate the EC_50_s of antiviral drugs. For these experiments, we decided to use cidofovir, which is a topical nucleoside analogue commonly used to treat FHV-1-induced ocular disease with reported clinical efficacy based on a controlled *in vivo* experimental study (12, 24). We and others have previously determined the EC_50_ of cidofovir for FHV-1 by using traditional plaque reduction assays and found the EC_50_ to range between 7.9 and 21.5 µM (25–27). To determine the EC_50_ by ECIS, CRFK cells were infected with FHV-1 at an MOI of 0.01 and treated with decreasing concentrations of cidofovir at the time of infection. Impedance changes were monitored over time until cell death was observed in all of the wells (Fig. 4A). The normalized impedance curves (Fig. 4A, insert) were used to calculate the Z′_50_ values for each cidofovir treatment, and these values were then used to construct a dose-response curve. The dose-response curve allowed us to compute an EC_50_ of 26.5 ± 9.9 µM. We found no statistically significant difference between the EC_50_s obtained by ECIS and those obtained by conventional plaque reduction assays (Student *t* test, *P* = 0.13, Fig. 4C), indicating that ECIS can be used to accurately determine the EC_50_ of antivirals. Finally, we determined the half-maximal cellular cytotoxicity (CC_50_) by using both ECIS and the conventional 3-(4,5-dimethyl-2-thiazolyl)-2,5-diphenyl-2H-tetrazolium bromide (MTT) cell viability assay. CC_50_s of 1,273 ± 124.7 and 1,600 ± 97.8 µM were calculated with the MTT assay and ECIS, respectively (see Fig. S3A and B), and although these values were in the same range (between 1,000 and 2,000 µM), they were found to be statistically significantly different (see Fig. S3C).

10.1128/mSphere.00039-17.3 FIG S3  Evaluation of cidofovir cytotoxicity. (A) Calculation of cidofovir CC_50_ at 5 days posttreatment by MTT cell viability assay. (B) Calculation of cidofovir CC_50_ at 3 days posttreatment by ECIS. Dotted lines represent the CC_50_ values. (C) Comparison of the CC_50_ values determined by the two methods. *, *P* < 0.05. Download FIG S3, PDF file, 0.2 MB. Copyright © 2017 Pennington and Van de Walle. 2017 Pennington and Van de Walle This content is distributed under the terms of the Creative Commons Attribution 4.0 International license.

## DISCUSSION

Traditionally, plaque assays or PCRs are used to evaluate single-step and multistep growth kinetics of herpesviruses, specifically focusing on factors related to viral entry and cell-to-cell spread, respectively (28). Here, we propose ECIS as a novel tool with which to study herpesvirus growth kinetics, with the major advantages that ECIS provides objective quantification in real time, thus avoiding the need for static intermittent sample collection and extensive postexperimental processing. ECIS specifically measures nanoscale morphological changes in infected cells and thus is a more sensitive tool than assessment of morphology by light microscopy or by fixing and staining infected cells (1). Here, we demonstrate the utility of ECIS for three specific virological applications. ECIS is likely to be most useful as a supplement to conventional assays or as an initial screening tool before further experimentation. With regard to the latter, ECIS allows the use of a 96-well plate format, which significantly increases the number of samples that can be run simultaneously compared to the conventional viral infectivity methodology and thus may provide a useful medium- to high-throughput platform for screening purposes related to herpesvirus growth and cellular morphological changes in response infection. Indeed, by our calculation for one 96-well plate, including appropriate controls and replicates, up to 45 individual samples can be run simultaneously.

When taking a closer look at the normalized ECIS data, we consistently observed an early spike in impedance following infection with FHV-1 at different MOIs, and this was also observed when measuring resistance and capacitance, the latter showing a decreased spike. These fluctuations can partially be explained by the physical manipulation of the cells required to add the virus or control medium, which results in temperature and pH changes, as well as the introduction of shear force. Indeed, a small spike is typically observed following any manipulation of wells in an ECIS experiment and has previously been described in the context of IAV infection (8). However, infection of cells with FHV-1 at high MOIs (i.e., 10 and 1) induced a spike in impedance and resistance, and a drop in capacitance, much larger than a small spike due to manipulation of the wells. To explore the underlying mechanism of these large fluctuations early after infection, we performed several experiments aimed at evaluating changes in the (i) shape, (ii) movement, and (iii) size of FHV-1-infected cells (data not shown). No dramatic rearrangements of the cytoskeleton of infected cells at early time points postinfection were observed, as assessed by immunofluorescence staining with fluorescently labeled phalloidin, which stains actin, suggesting that large-scale morphological changes are not the reason for the large spikes. Live-cell imaging revealed migration of infected cells starting at several hours postinfection, which does not correspond to the time point early after infection when the spikes were recorded by ECIS. Finally, we also evaluated cell size with an established flow cytometry assay (29) based on previous work showing that HSV-1 can induce swelling of infected cells early after infection (30). Using flow cytometry, we did not observe an increase in cell size following infection of cells with high MOIs of FHV-1 in the first 4 h of the time course, but in contrast, and to our surprise, we actually observed a decrease in cell size. As ECIS can quantify minute changes in cell shape, size, and movement (1), it is possible that these more conventional methodologies are not sufficiently sensitive to report the changes that we detect via ECIS. Likewise, ECIS analyses of infection with IAV has reported a similar observation consisting of fluctuations in impedance immediately following infection (8), but without a full understanding of the biological implications underlying this phenomenon, it remains elusive what exactly causes these ECIS changes.

In our present study, we used the ZƟ instrument from Applied BioPhysics, which is a more recent ECIS system that allows the direct measurement of resistance, representing cell-to-cell interactions, and capacitance, representing cell-to-substrate interactions. Previous models determined impedance by using Ohm’s law and were therefore unable to report these values. These individual measurements are valuable, as they provide more biological information about the different cellular responses to infection than impedance analyses alone. In our study with FHV-1, the rates of change in resistance and capacitance were practically identical, indicating that FHV-1 most likely induces structural changes in infected cells, resulting in reduced cell-to-cell contacts and detachment from the culture plate simultaneous. It will be interesting to evaluate these different parameters in cells infected with other viruses to get a better idea of the biological relevance of these findings in virus-infected cells beyond the simple formation of a CPE. Furthermore, the ZƟ instrument allows easy calculation of barrier resistance (Rb), alpha, and membrane capacitance (Cm) values (31). Rb describes resistivity, an intrinsic property that quantifies how strongly a given material opposes the flow of electrical current at cell-to-cell contacts and therefore provides information about the permeability of the monolayer to electrical current flow. Rb is commonly used to assess the monolayer integrity of endothelial cells. Alpha is a measure of the constraint on current flow beneath the cells and thus describes changes in the region beneath the cells, and Cm represents the average capacitance of the cell plasma membranes (32–34). However, a tight cellular monolayer is essential in order to accurately model these values and since the CRFK cells used in our study did not form a tight enough monolayer, even after several days of growth to the point of overconfluence (data not shown), were we unable to model these values. However, other cell lines commonly used for viral infectivity assays, such as Madin-Darby canine kidney (MDCK) cells, do produce sufficiently tight monolayers to allow modeling of these values (33). Infecting MDCK cells with the alphaherpesvirus canine herpesvirus type 1, which is closely related to FHV-1 and also a causative agent of an ocular disease similar to HSV-1-induced ocular disease in humans (35), could be used in future ECIS experiments to evaluate these values and determine their biological importance in alphaherpesvirus infections.

We did find a statistically significant difference between the CC_50_ values calculated by ECIS and the conventional MTT assay. The higher CC_50_ calculated by ECIS is likely due to a difference in the measured variable between the two assays. MTT assays measure the conversion of the substrate to formazan crystals in the mitochondria and thus measure the very early stages of cell death (36, 37). In comparison, ECIS measures morphological changes, more specifically, the rounding of cell membranes and detachment from the substrate, which corresponds to later stages of cell death.

Taken together, our data show that ECIS, in conjunction with current methodologies, can be a powerful and valuable complementary tool with which to monitor viral growth and study the cellular response to alphaherpesvirus infection.

## MATERIALS AND METHODS

### Virus, cells, and antiviral drug.

For this study, FHV-1 strain FH2CS was used (38). CRFK cells (American Type Culture Collection) were maintained in a cell line medium consisting of Dulbecco’s minimal essential medium (DMEM) with 1 g/liter glucose, l-glutamine, and sodium pyruvate, 10% fetal bovine serum, and penicillin (200 U/ml)-streptomycin (200 μg/ml) and cultured at 37°C and 5% CO_2_. The nucleoside analogue cidofovir, available as a 75-mg/ml intravenous solution (Vistide; Gilead Sciences, Foster City, CA), was used at concentrations ranging between 1.9 and 976 μM in the EC_50_ experiment and between 4.9 and 5,000 µM in the CC_50_ experiment.

### ECIS.

ECIS was used to monitor virus- or drug-induced cellular changes as a proxy for cell death in a variety of experiments. To this end, 20,000 CRFK cells were plated into triplicate wells of a 96W10idfPET plate (Applied BioPhysics Inc., Troy, NY) that had been pretreated for 15 min with 10 mM l-cysteine at room temperature (RT), followed by 30 min with cell line medium. The plate was allowed to rest for 30 min at RT prior to incubation and ECIS monitoring to allow even distribution of the cells in the wells. Cells were infected at 24 hpp with FHV-1 at different MOIs ranging from 0.0001 to 10 and treated with or without the antiviral cidofovir at different concentrations, depending on the experiment. Mock-infected and cell-free wells were included as controls for all experiments. Capacitance (C) and resistance (R) were measured at the indicated frequencies in a series RC circuit for 24 h with an ECIS Model Zϴ instrument with a 96-well array station (Applied BioPhysics Inc., Troy, NY), and these values were then used to automatically calculate complex impedance (Z). Measurements were taken at the minimal time interval allowed by the plate setup (typically every 5 to 12 min, depending on the number of samples run in an experiment). An additional ECIS 96W10idfPET plate was similarly prepared, and images were captured at specified intervals with an Olympus CKX41 microscope (Olympus, Center Valley, PA) controlled with Infinity Analyze version 6.4 software (Lumenera Corporation, Ottawa, Ontario, Canada) for microscopic analyses.

### ECIS data analyses.

To determine the appropriate frequencies at which to evaluate impedance (Z), resistance (R), and capacitance (C), the ratio of cell-containing wells to cell-free wells for each parameter was plotted as a function of frequency at 24 hpp, prior to addition of the virus. The frequency with the greatest difference between the cell-free and sample wells for each parameter was used as the optimal frequency for all further experiments.

Impedance data were normalized (Z′) by using the formula *Z*′ = (*Z*_*x*_ − *Z*_end_)/(*Z*_0 hpi_ − *Z*_end_) to normalize the starting impedance at the time of infection (*Z*_0 hpi_) to a value of 1 and the final impedance at the end of the experiment (*Z*_end_) to a value of 0, scaling the impedance of the intervening time points (*Z*_*x*_) proportionally. The same formula was also used to normalize resistance (*R*′). To normalize capacitance (C′), the formula *C*′ = (*C*_*x*_ − *C*_0 hpi_)/(*C*_end_ − *C*_0 hpi_) was used to normalize the starting capacitance at the time of infection (*C*_0 hpi_) to a value of 0 and the final impedance at the end of the experiment (*C*_end_) to a value of 1, scaling the intervening time points (*C*_*x*_) proportionally. GraphPad Prism (version 6.04 for Windows) was used to fit the normalized data for each of the three parameters into a sigmoidal, four-parameter logistic dose-response curve with a least-squares fit model, and the half-maximal normalized impedance (Z′_50_), resistance (R′_50_), or capacitance (C′_50_) was calculated on the basis of this curve. To determine the EC_50_ of cidofovir, a dose-response curve was constructed by using the Z′_50_ values and the EC_50_ was calculated similarly.

### Creation of DsRed-labeled FHV-1.

FHV-1-gD-DsRed was created by CRISPR/Cas9 genome engineering on the basis of previously described protocols for HSV-1 editing (18–20). Briefly, the CRISPR plasmid was engineered by the method described by Ran et al. (19). The sgRNA Forward and sgRNA Reverse primers (Table 1) were annealed together and cloned into the pSpCas9(BB)-2A-Puro (PX459) v 2.0 vector, a gift from Feng Zhang (Addgene plasmid 62988) (see Fig. S2A). A donor plasmid created to drive insertion of the DsRed Express2 gene into the C-terminal end of *US6* by homology-directed repair consisted of a 620-bp virus fragment corresponding to the C terminus of *US6*, the 675-bp DsRed Express2 gene, and a 605-bp virus fragment corresponding to the *US6* stop codon, the intergenic region between *US6* and *US7*, and the initial part of the *US7* gene cloned into the pJET1.2 PCR cloning vector (Thermo Fisher Scientific, Waltham, MA) (see Fig. S2B). The CRISPR/Cas9 and donor plasmids (1,250 ng of each) were transfected into confluent CRFK cells with LT1 transfection reagent for 3 days (Mirus Bio LLC, Madison, WI). Transfected cells were selected with 5 µg/ml puromycin in cell line medium for 3 days and allowed to recover for 1 week. Next, cells were transfected with an additional 500 ng of donor plasmid and simultaneously infected with approximately 6,500 PFU of FHV-1. A pure FHV-1-gD-DsRed stock was then obtained by three rounds of limiting-dilution assays (28).

**TABLE 1:** Primers used to create single guide RNAs (sgRNAs), a donor plasmid, and a qPCR plasmid standard

Primer name	Sequence (5'-3')	Description
sgRNA Forward	CACCGTTGGAATGTGGACTTAAGGA	sgRNA to *US6* stop codon
sgRNA Reverse	AAACTCCTTAAGTCCACATTCCAAC	sgRNA to *US6* stop codon
Primer 1	CGGCCCAATTTAATCAAGG	*US6* homology arm, forward
Primer 2	AGGATGGTGAGTTGTATGTA	*US6* homology arm, reverse
Primer 3	GTCCACATTCCAATCGAGTT	*US7* homology arm, forward
Primer 4	AACACCGAAAGGCCAAATAC	*US7* homology arm, reverse
Primer 5	ATGGATAGCACTGAGAACGT	DsRed Express2, forward
Primer 6	TTACTGGAACAGGTGGTGGC	DsRed Express2, reverse
Primer 7	ACTCACCATCCTATGGATAGCACT	*US6*/DsRed overhang, forward
Primer 8	TGGAATGTGGACTTACTGGAACAG	*US7*/DsRed overhang, reverse
Primer 9	AGTGCTATCCATAGGATGGTGAGT	*US6*/DsRed overhang, reverse
Primer 10	CTGTTCCAGTAAGTCCACATTCCA	*US7*/DsRed overhang, forward
*US7* plasmid standard forward	CTTTCCGGTCCTGTCTCCAC	qPCR forward primer
*US7* plasmid standard reverse	GGTTAAATCTTACCCGCAGTGC	qPCR reverse primer

Insertion of DsRed into the desired location in FHV-1 was verified by traditional PCR and Sanger sequencing at the Cornell University Institute of Biotechnology. Immunofluorescence microscopy was used to verify the expression of the DsRed protein in FHV-1-gD-DsRed-infected CRFK cells, which were counterstained with a mouse anti-FHV-1 monoclonal antibody (clone FHV7-7C; Bio-Rad, Hercules, CA) and 4',6-diamidino-2-phenylindole (DAPI). WT FHV-infected CRFK cells and isotype control antibodies (Abcam, Inc., Cambridge, United Kingdom) were included as controls. Cells were imaged exactly as previously described (27).

### Evaluation of FHV-1-gD-DsRed growth kinetics by conventional viral plaque assays.

Viral plaque size assays were performed exactly as previously described (39), and 50 plaques were captured with an Olympus CKX41 microscope (Olympus, Center Valley, PA) controlled with Infinity Analyze version 6.4 software (Lumenera Corporation, Ottawa, Ontario, Canada). ImageJ was used to measure the area of each plaque.

Single-step (MOI of 3) and multistep (MOI of 0.01) growth kinetic assays were performed, also exactly as previously described (39), except that the adsorption period lasted 2 h. Samples were collected at the intervals indicated with cell-free supernatant samples used for standard plaque assays (27) and cell lysates used for quantitative PCR (qPCR). For the latter, a standard curve with primers targeting a region in the *US7* gene homology arm was created with the linearized donor plasmid used to create FHV-1-gD-DsRed as the template (Table 1). Efficiency of amplification was confirmed to be >98% with *R*^2^ = 0.998, and qPCR was performed as previously described (28). For samples containing large amounts of virus, template DNA was first diluted 1:100 to keep the copy number within the limits of the standard curve. The standard curve was used to interpolate the genome copy numbers, which were expressed in genomes per cell on the basis of the estimation that 5,000 cells contain approximately 30 ng of DNA (40).

### Cidofovir toxicity assays.

The MTT assay was used to assess cidofovir toxicity in a manner similar to that previous described (41, 42). Briefly, 20,000 cells were plated into duplicate wells of a 96-well plate. Cells were treated with 2-fold serial dilutions of cidofovir at 24 hpp. At 5 days posttreatment, MTT dissolved in DMEM was added to cells for 1 h of incubation. The resulting formazan crystals were dissolved in an equal volume of the solubilization solution. The absorbance at 570 nm was then measured spectrophotometrically and used to construct a dose-response curve, and the CC_50_ was determined. Cidofovir toxicity was evaluated by ECIS in a manner similar to that previous described (43, 44). Briefly, 20,000 CRFK cells were plated and monitored for 24 h as described above. Cells were then treated with 2-fold serial dilutions of cidofovir. At 3 days posttreatment, the CC_50_ was calculated on the basis of the normalized impedance.

### Statistical analyses.

Data were statistically evaluated by GraphPad Prism (version 6.04 for Windows) and are expressed as the mean ± standard deviation. On all ECIS graphs, the mean is presented as a solid line and standard deviations are presented as dotted lines. For comparisons of the Z′_50_, R′_50_, and C′_50_ values across infections with different MOIs, one-way ANOVAs were performed, followed by Tukey’s honest significant difference test, to establish significance for the multiple comparisons. For the plaque size assays, normality was first assessed with the Shapiro-Wilks test and significance was then established with a Mann-Whitney U test. All other statistical analysis was performed with Student *t* tests. All experiments were performed three times. ECIS experiments were additionally performed with three technical replicates per sample. A *P* value of <0.05 was considered significant.
