# Supplementary figures and images for: Electric Cell-Substrate Impedance Sensing To Monitor Viral Growth and Study Cellular Responses to Infection with Alphaherpesviruses in Real Time
Source: mSphere. 2017 Apr 5;2(2):e00039-17. doi: 10.1128/mSphere.00039-17 (PMC5381265; doi:10.1128/mSphere.00039-17)

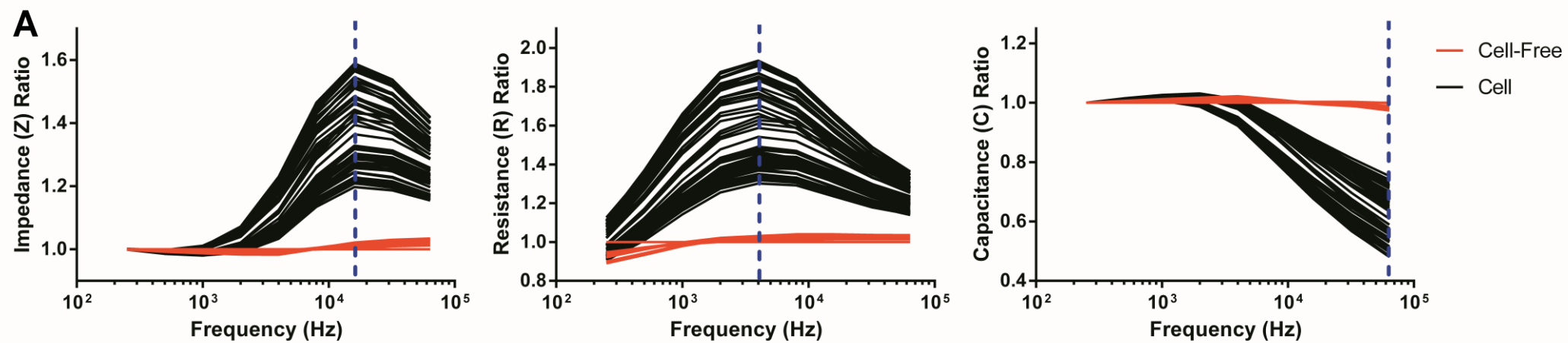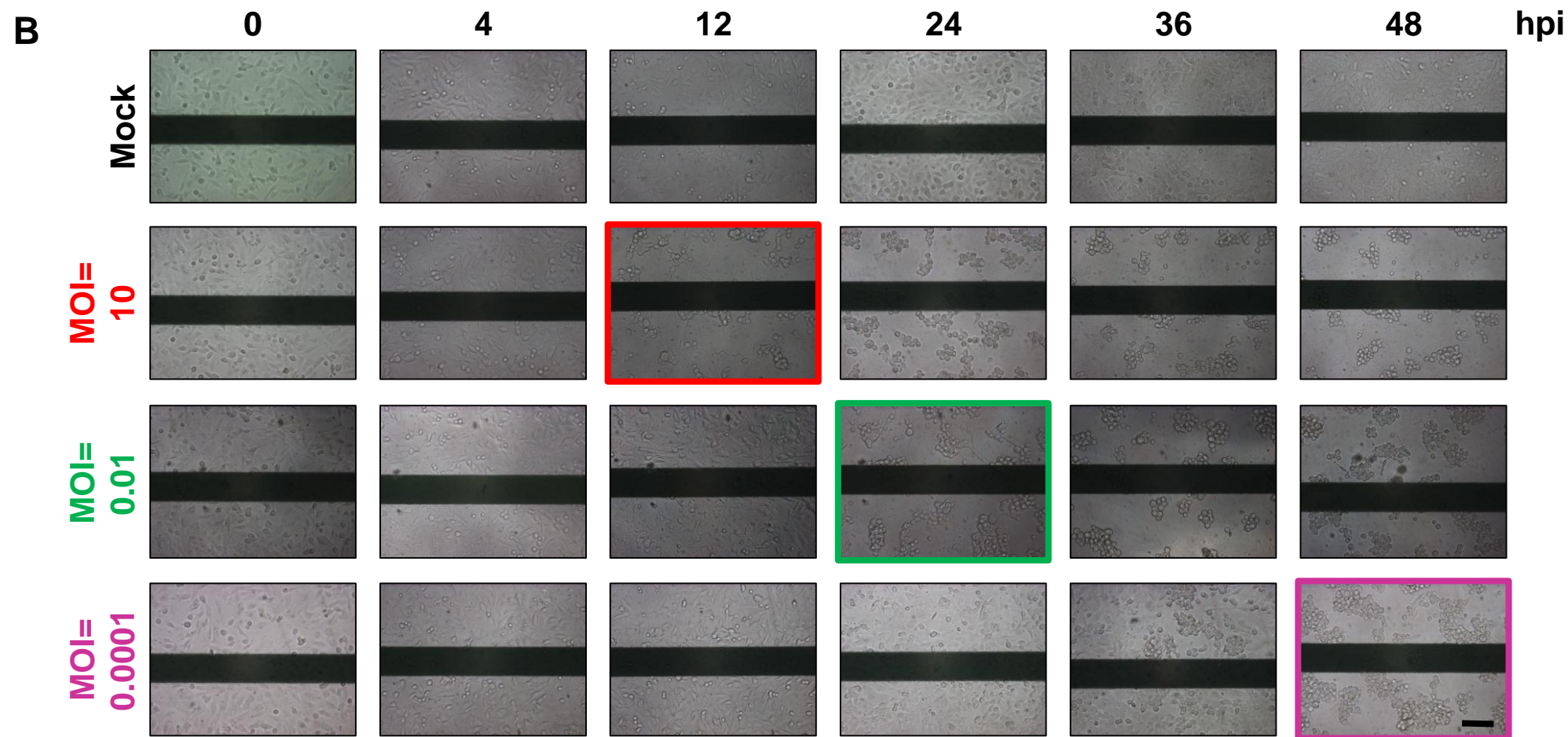

Supplement: FIG S1 [file sph002172265sf1.pdf]

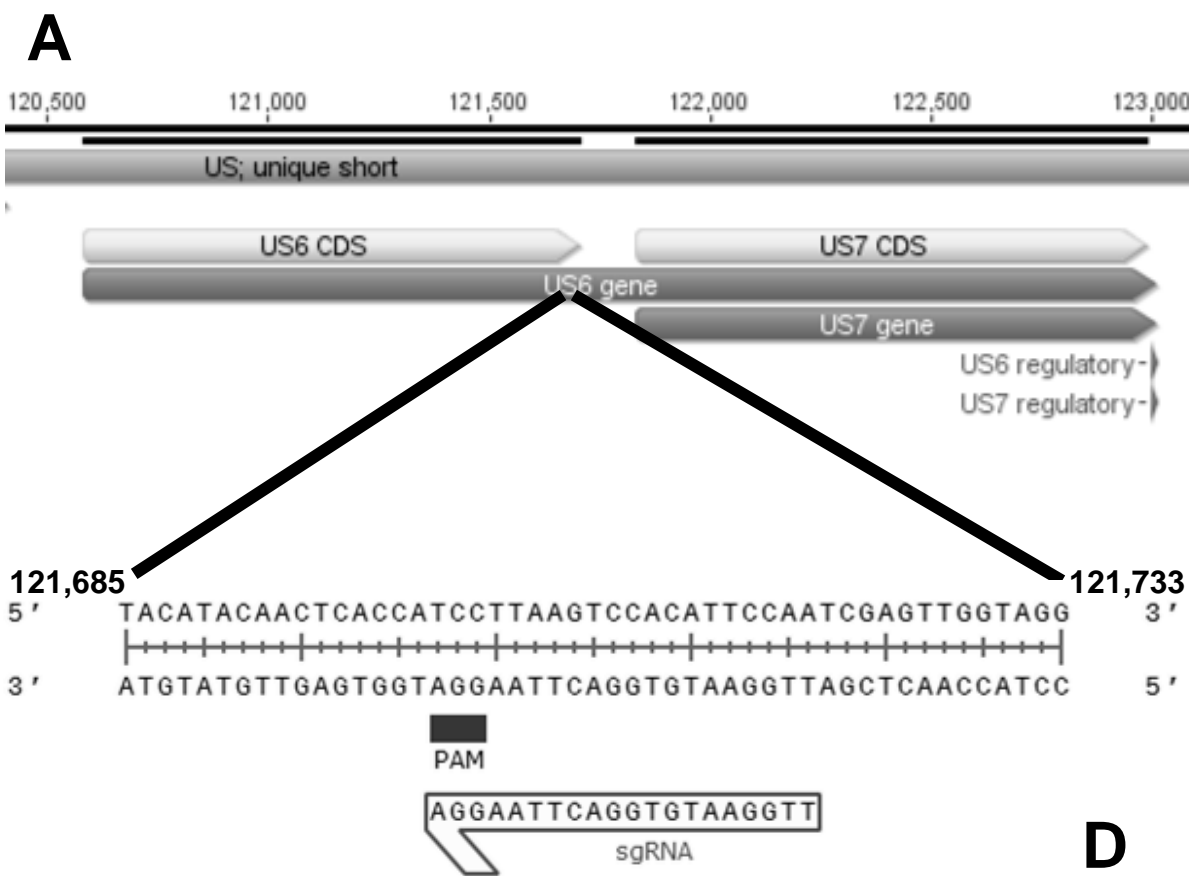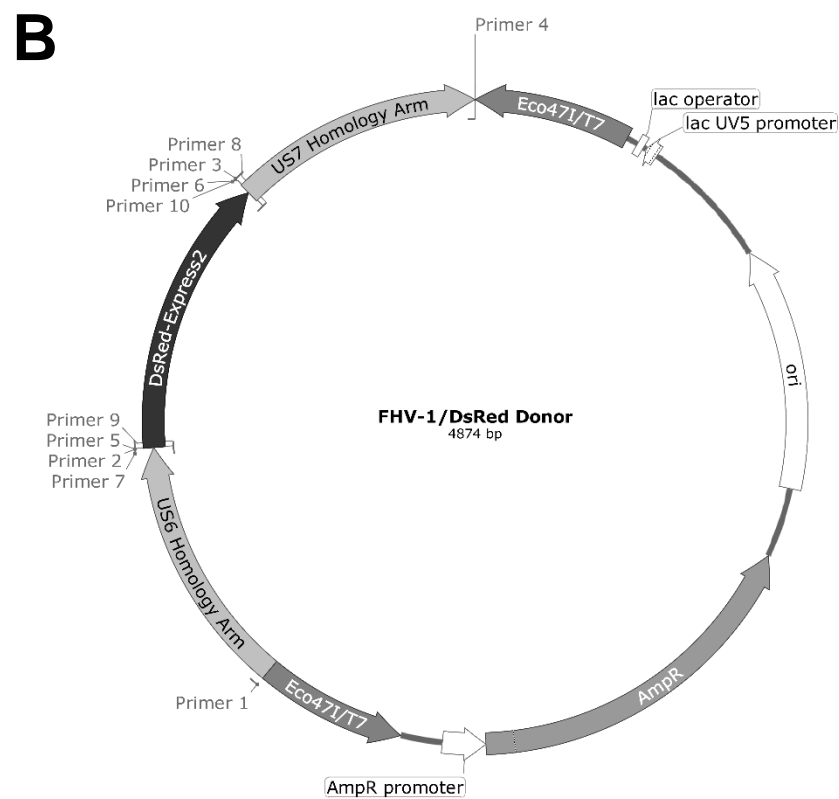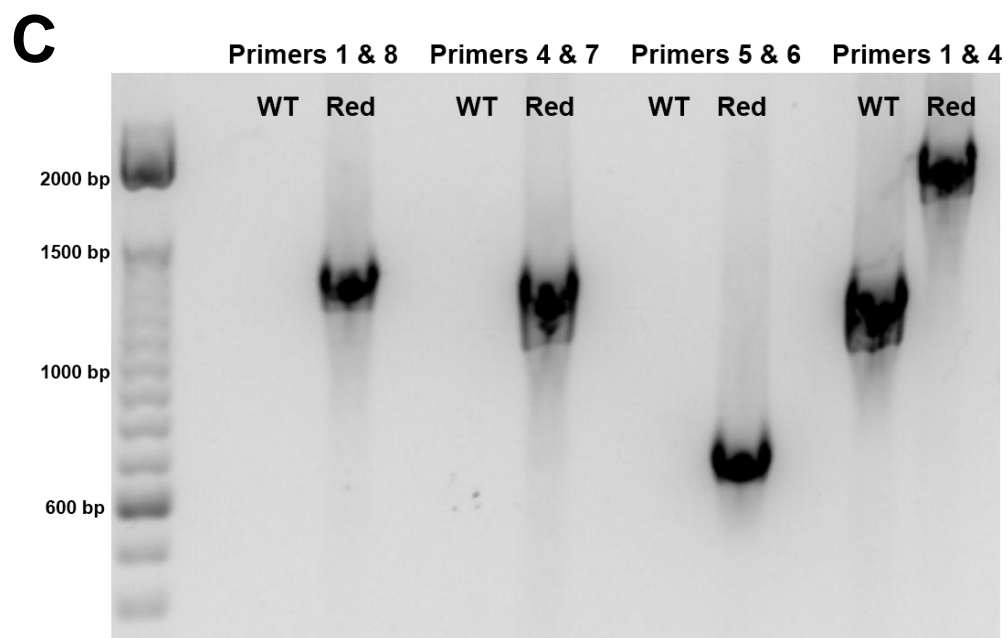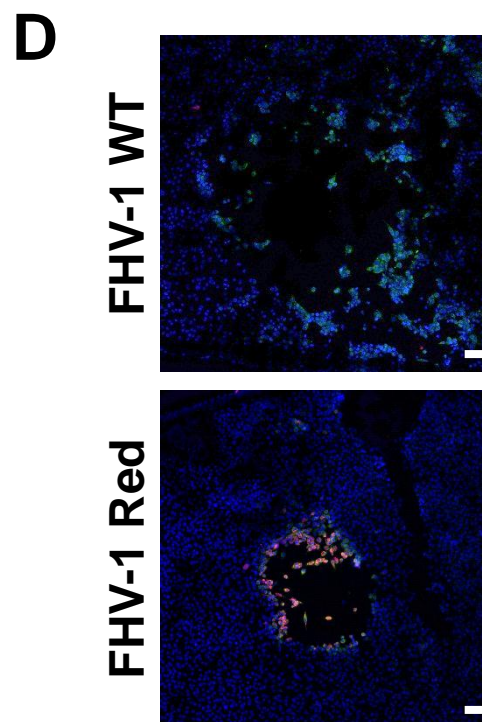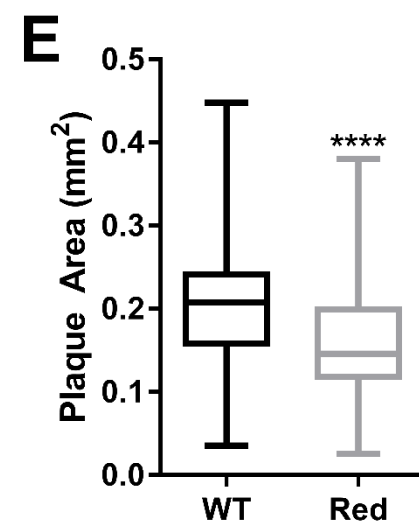

Supplement: FIG S2 [file sph002172265sf2.pdf]

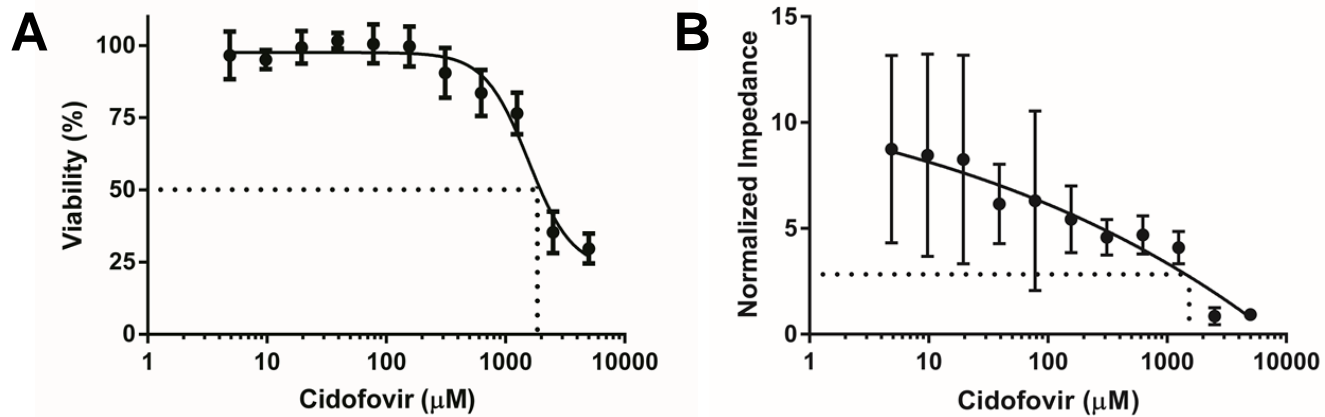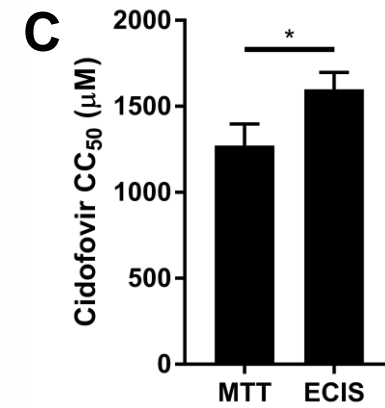

Supplement: FIG S3 [file sph002172265sf3.pdf]
